# An emerging and variant viral promoter of HIV-1 subtype C exhibits low-level gene expression noise

**DOI:** 10.1186/s12977-021-00572-2

**Published:** 2021-09-19

**Authors:** Haider Ali, Disha Bhange, Kavita Mehta, Yuvrajsinh Gohil, Harshit Kumar Prajapati, Siddappa N. Byrareddy, Shilpa Buch, Udaykumar Ranga

**Affiliations:** 1 Molecular Biology and Genetics Unit, HIV AIDS Laboratory, Jawaharlal Nehru Centre for Advanced Scientific Research, Jakkur, Bangalore, 560064 India; 2 Department of Pharmacology and Experimental Neuroscience, University of Nebraska Medical Center, Omaha, NE USA

**Keywords:** HIV-1, Subtype C, Transcriptional silence, LTR, Latency reversal, Sequence duplication

## Abstract

**Background:**

We observe the emergence of several promoter-variant viral strains in India during recent years. The variant viral promoters contain additional copies of transcription factor binding sites present in the viral modulatory region or enhancer, including RBEIII, LEF-1, Ap-1 and/or NF-κB. These sites are crucial for governing viral gene expression and latency. Here, we infer that one variant viral promoter R2N3-LTR containing two copies of RBF-2 binding sites (an RBEIII site duplication) and three copies of NF-κB motifs may demonstrate low levels of gene expression noise as compared to the canonical RN3-LTR or a different variant R2N4-LTR (a duplication of an RBEIII site and an NF-κB motif). To demonstrate this, we constructed a panel of sub-genomic viral vectors of promoter-variant LTRs co-expressing two reporter proteins (mScarlet and Gaussia luciferase) under the dual-control of Tat and Rev. We established stable pools of CEM.NKR-CCR5 cells (CEM-CCR5_RL_ reporter cells) and evaluated reporter gene expression under different conditions of cell activation.

**Results:**

The R2N3-LTR established stringent latency that was highly resistant to reversal by potent cell activators such as TNF-α or PMA, or even to a cocktail of activators, compared to the canonical RN3- or the variant R2N4-LTR. The R2N3-LTR exhibited low-level basal gene expression in the absence of cell activation that enhanced marginally but significantly when activated. In the presence of Tat and Rev, trans-complemented in the form of an infectious virus, the R2N3-LTR demonstrated gene expression at levels comparable to the wild-type viral promoter. The R2N3-LTR is responsive to Tat and Rev factors derived from viral strains representing diverse genetic subtypes.

**Conclusion:**

With extremely low-level transcriptional noise, the R2N3-LTR can serve as an excellent model to examine the establishment, maintenance, and reversal of HIV-1 latency. The R2N3-LTR would also be an ideal viral promoter to develop high-throughput screening assays to identify potent latency-reversing agents since the LTR is not affected by the usual background noise of the cell.

## Background

The human immunodeficiency virus type-1 (HIV-1) is classified into several subtypes based on genetic heterogeneity, and the global distribution of the viral subtypes is uneven [1]. Of the various genetic subtypes, subtype C of HIV-1 (HIV-1C) is responsible for nearly half of global HIV-1 infections and the vast majority of infections of India. While several differences, including the demographic factors, host factors, and founder effect, may contribute to differential distribution properties of closely related strains of pathogenic organisms, unique biological properties intrinsic to different genetic groups may play a crucial role. Several molecular features uniquely or preferentially associated with HIV-1 genetic subtypes have been mapped to viral regulatory elements, including the promoter, structural, regulatory, and accessory proteins of the virus [1].

The long-terminal repeat (LTR) of the viral subtypes is highly diverse, differing up to 20–25% among subtypes [2, 3]. Although the basic domain structure of the viral promoter is preserved among viral subtypes, subtype-associated differences within each transcription factor binding site (TFBS) are evident. For example, HIV-1C manifests several distinct differences in the composition of the TFBS, including NF-κB [4, 5], NF-AT, upstream stimulatory factor (USF) [6], and other regulatory elements such as the TATA box and the TAR region [7]. Of these LTR variations, subtype-specific differences within the enhancer element, exclusively consisting of the NF-κB motifs, are crucial given the profound impact NF-κB has on viral gene expression (8). HIV-1C LTR contains not only more copies of the NF-κB motif (3 or 4 motifs) but the additional copies are also genetically diverse. A positive correlation has been demonstrated between the number of NF-κB motifs and the transcriptional activity of the LTR [9]. Thus, compared to the other viral subtypes, the enhancer element in HIV-1C is characterized by a greater variation in terms of κB-site number and sequence variation.

In addition to the NF-κB site variation described above, the duplication of the RBEIII motif, an upstream element located in the modulator of the viral promoter, is crucial given the significance of this motif to viral latency [10]. The duplication of the RBEIII motif, a binding site for the RBF-2, popularly known as the most frequent naturally occurring length polymorphism (MFNLP), has been found in approximately 38% of HIV-1B-infected subjects [11]. The functional significance of the MFNLP has been extensively and exclusively investigated in the context of HIV-1B [12], although the inferences drawn from these studies are inconclusive.

A recent work from our laboratory shows the emergence of several promoter-variant strains in India [13]. In collaboration with four different clinical sites in India, we screened more than 750 primary viral strains. We found the emergence of at least nine variant viral strains during the past decade. The promoter-variant strains contain additional copies of TFBS present in the enhancer and/or the modulatory region of the LTR, created by sequence duplication, including those of NF-κB, RBEIII, LEF-1 and Ap-1. The master regulatory circuit of HIV-1 comprising the LTR and Tat controls viral gene expression and transcriptional silence. The specific composition of the TFBS in the emerging viral variants could significantly impact on the nature of viral gene expression. Preliminary data evaluating gene expression from a few specific variant viral LTRs, especially those containing the duplication of NF-κB and/or RBEIII motifs, and encoding reporter genes, show that viral latency establishment, maintenance, and reversal properties are significantly modified.

The variants of concern are referred to as ‘double RBEIII’ or ‘RR’ variants and contribute to 18.7% of all the promoter variants identified in the clinical study [13]. The R2N3-LTRs constitute 3.3% of all the promoter variants. The analysis of prognostic markers has also been presented for RR variants in Fig. 4 of Bhange D et al., [13]. R2N3-LTR, a variant viral promoter containing two RBEIII motifs (RBEIII motif duplication) and three NF-κB sites (wild-type-like configuration) can establish latency at a rapid rate. Of note, the R2N3-LTR maintains avid latency in Jurkat cells and primary CD4^+ve^ cells that is hard to reverse as compared to the RN3-LTR, the canonical wild-type HIV-1C promoter containing a single RBEIII motif and three NF-κB sites or R2N4-LTR, a different variant promoter containing the co-duplication the RBEIII and NF-κB motifs [13]. Host factors binding to the RBEIII site in HIV-1 LTR in conjunction with c-Jun can regulate the establishment and maintenance of latency [14]. In the R2N3-LTR, the presence of an additional copy of the RBEIII motif, especially in the absence of the co-duplication of the NF-κB site (contains only three copies of NF-κB motifs) makes the viral promoter hard-to-reverse from latency. Notably, the R2N3-LTR is characterized by significantly low transcriptional noise compared to RN3- or R2N4-LTRs.

In summary, several TFBS-variant viral strains have been emerging in HIV-1C in recent years. The specific configuration of the TFBS profile of the variant viral strains appears to impact gene expression, transcriptional noise, and latency properties of the viral promoter. Here, we evaluate and compare viral gene expression from a panel of variant viral promoters. We constructed sub-genomic viral vectors co-expressing two different reporter genes, mScarlet (a red fluorescent protein (RFP)) and Gaussia Luciferase (G-Luc), subject to Tat and Rev co-expression. Further, we established CEM-CCR5_RL_ stable cell pools and compared viral proliferation properties of diverse HIV-1 molecular clones and primary viral strains under different conditions of cell activation. The results show that the R2N3-LTR differs significantly from the wild-type RN3- or variant R2N4-LTRs in demonstrating significantly low levels of transcriptional activity.

The Tat and Rev dependent lentiviral vectors offer an additional layer of specificity as they are also Rev-dependent in addition to Tat-dependence. The HIV-1 Rev protein binds to the Rev responsive element (RRE) of HIV-1 RNA and exports the RRE containing unspliced RNA from the nucleus to the cytoplasm [15]**.** The Rev/RRE functional activity may vary depending on subtype-specific characteristics [16]. To this end, we attempted to characterize subtype-specific activities of Rev and RRE combinations to generate an efficient Tat- and Rev-dependent reporter vector. Here, we evaluated subtype-specific activities of Rev-RRE combinations, in addition to examining the validity of a few emerging and variant HIV-1C viral promoters as reporter vectors.

The primary objective of the present work is to explore the potential of R2N3-LTR as a reporter vector to monitor HIV-1 infection. To this end, we employed three major experimental strategies. First, we assessed the transcription noise from different HIV-1 promoters. We compared the R2N3 and additional double-RBEIII promoter R2N4 with the canonical RN3 promoter. After confirming a significantly low background noise from R2N3, we explored the potential of this LTR as a reporter. Second, we attempted to optimize the reporter system to further improve it. Most of the reporter cell lines available for HIV-1 infection are of HIV-1B origin. Since a reporter system of HIV-1C origin is being used here, we desired to optimize the other regulatory elements, such as the RRE. Finally, we constructed and evaluated the optimized reporter system. The optimized vector comprised a combination of the R2N3-LTR and HIV-1C RRE.

## Results

### Generation of a panel of Tat- and Rev-dependent reporter lentiviral vectors and CEM.NKR-CCR5 stable cell pools

We aimed to examine the gene expression properties of R2N3-LTR, a unique HIV-1 variant promoter that has been emerging in recent years in India.

**Fig. 1:**
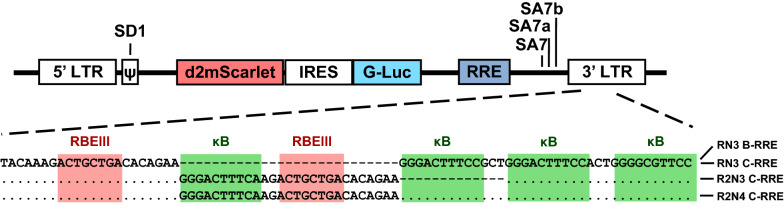
RBEIII duplication reduces background noise. Schematic representation of a panel of Tat- and Rev-dependent dual-reporter expression (mScarlet and G-Luc) lentiviral vectors. mScarlet (d2mScarlet) contains a degradation domain. The reporter gene expression cassette and the RRE motif are flanked by the splice donor and acceptor elements. The NF-κB motifs are highlighted by green background color and the RBEIII sites by red color. Dashes in the multi-sequence alignment indicate sequence deletion and dots sequence identity. N and R represent the NF-κB and RBF-2 transcription factor binding sites, respectively

We constructed a panel of four sub-genomic viral strains comprising of three LTRs and two Rev-responsive elements (RRE). The panel consisted of the canonical RN3-LTR containing one RBEIII motif and three NF-κB sites, the variant R2N3-LTR containing an additional RBEIII motif, and a different variant promoter R2N4-LTR containing a co-duplication of an NF-κB motif and an RBEIII site (Fig. 1). All the viral strains co-expressed two different reporter genes. The expression of mScarlet (RFP) was placed directly under the control of the viral promoter. The RFP contained a degradation domain that reduced the protein half-life to approximately 2 h [17]. A reporter protein with a short half-life would faithfully represent the transcriptional status of the viral promoter. Additionally, the expression of G-Luc was placed under the control of an internal ribosomal entry site (IRES) element. G-Luc is naturally secreted from the cell into the culture medium, thus offering a convenient and non-invasive assay for the transcriptional activity of the viral promoter. Notably, the expression cassette flanked by the major splice donor SD1 and splice acceptor site SA7 will be removed in the absence of HIV-1 Rev, thus making the expression of the two reporter genes dependent on Rev. Additionally, given that the function of the LTR is dependent on Tat transactivation, the reporter gene expression is conditional to the presence of both Tat and Rev proteins [14, 18, 19].

Using this expression system, we compared the functioning of the two variant viral LTRs, R2N3 and R2N4, with the canonical HIV-1C promoter RN3 under different conditions. Viral stocks of all the three viral strains were prepared in HEK293T cells by pseudotyping the viral particles with a VSV-G envelope using the standard calcium chloride transfection procedure [20].

CEM.NKR-CCR5 human lymphoblastoid T cell line stably expressing the CCR5 chemokine receptor was chosen for the generation of stable cells. CEM.NKR-CCR5 cells express the CD4 receptor necessary for the virus to infect the target cells. Additionally, the cells also constitutively express the CXCR4 and CCR5 co-receptors. CEM.NKR-CCR5 cells, therefore, can be permissive for productive infection of both R5- and X4-tropic HIV-1 viral strains. CEM.NKR-CCR5 cells were independently infected with the three members of the viral panel at a multiplicity of infection (MOI) of 0.02 to 0.04, and cells stably expressing RFP, referred to as CEM-CCR5_RL_, were selected by flow sorting.

### Differential activation properties of the variant viral promoters

HIV-1 viral promoter is unique in containing binding sites for several families of transcription factors; often, these sites are present in multiple copies arranged in tandem. Typically, several of these sites overlap with each other. The promoter is functional at the basal level in the absence of Tat due to Tat-independent transcriptional activity regulated by transcription factors such as Sp1. The basal-level activity of the HIV-1 LTR can cause a significant magnitude of transcriptional noise. We surmised that the presence of an additional copy of NF-κB in the variant LTRs, R2N3, and R2N4, may quench the transcriptional noise as compared to the canonical RN3-LTR.

**Fig. 2:**
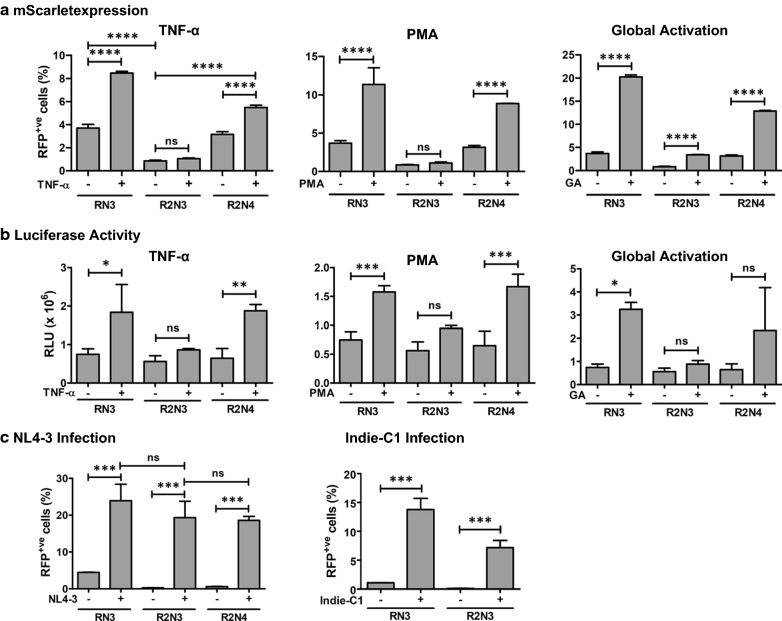
The transactivation profiles of the variant LTRs (**A**) RFP expression and (**B**) luciferase expression. CEM-CCR5_LR_ reporter cells were activated with TNF-α (10 ng/ml), PMA (5 ng/ml), or with a cocktail of four activators (10 ng/ml TNF-α, 5 ng/ml PMA, 100 nM TSA, and 2.5 mM HMBA). The expression levels of RFP were measured 24 h following activation. Each data point represents the mean of three replicate values. The data are presented as the mean percentage value ± SD of RFP positive cells from three independent experiments. We used ordinary one-way ANOVA with Sidak’s multiple comparison test (*ns* no significant, *p < 0.05, ***p < 0.05, ****p < 0.0001) for statistical analysis. **C** Transactivation profile following viral infection: cells were infected with NL4-3 viral strains as a source of Tat and Rev and the RFP expression levels were quantitated as described above. One-way ANOVA with Sidak’s multiple comparison test was used (*ns *no significant and ***p  < 0.05)

To understand such differential activity of the variant viral LTRs, we subjected pools of CEM-CCR5_RL_ cells representing the three members of the panel to different cell activation conditions in the absence of Tat and Rev (Fig. 2). The cells were activated for 24 h with TNF-α (10 ng/ml), PMA (5 ng/ml), or a cocktail of global activators (10 ng/ml TNF-α, 5 ng/ml PMA, 100 nM TSA, and 2.5 mM HMBA). After gating on live cells, we analyzed the cells for RFP expression by flow cytometry.

The canonical RN3-LTR showed a significant basal level activity without activation, which enhanced several folds following activation, as expected (Fig. 2). For example, 3.71 ± 0.32% of RN3 cells showed RFP expression in the absence of activation, which enhanced to 8.47 ± 0.15% cells following TNF-α activation, an enhancement of 2.3 folds which is statistically significant (p < 0.0001) (Fig. 2, Top panel). Comparable activation levels were observed with RN3-LTR following PMA or global activation, 3.10 and 5.46 folds of activation, respectively. Notably, the R2N4-LTR, which contained a co-duplication of both TFBS motifs, behaved the same way as the canonical RN3-LTR. This promoter demonstrated high levels of basal-level activity in the absence of activation, which enhanced 1.74, 2.81, and 4.08-folds following activation with TNF-α, PMA, and global activation, respectively, the differences being statistically significant (Fig. 2A). In contrast, the response of R2N3-LTR to the diverse activation conditions was quite different. R2N3-LTR showed low levels of basal activity in the absence of cellular activation. For example, only 0.85 ± 0.07% of R2N3 cells were positive for RFP in the absence of activation under the TNF-α panel, as compared to 3.71 ± 0.32 and 3.15 ± 0.24% of RN3 and R2N4 cells, respectively. These differences were statistically significant, ascertaining a low basal-level gene expression from R2N3-LTR compared to the other two promoters. Importantly, cellular activation with a single activator, TNF-α or PMA, failed to augment transactivation from R2N3-LTR, unlike in the case of the other two viral promoters. For instance, the basal level of 0.85 ± 0.07% of R2N3 cells increased only to 1.06 ± 0.06% following TNF-α activation, which is only a 1.24-fold enhancement, not statistically significant. However, under global activation conditions, mediated by a pool of four different activators, the percentage of RFP positive cells of R2N3 increased from 0.85 ± 0.07% to 3.40 ± 0.08% following activation, which is statistically significant (p < 0.0001). Comparable results were obtained by quantitating the luciferase levels secreted into the medium (Fig. 2B).

Of note, the above experiments were conducted in the absence of both Tat and Rev. To evaluate the effect of the presence of the two viral factors on the expression of the reporter genes; we infected the cells with replication-competent viral strain NL4-3 to serve as the source of Tat and Rev (Fig. 2C, left panel). The stringent quality of gene expression from the two LTRs containing the RBEIII motif duplication was evident from the data. The canonical RN3-LTR demonstrated 4.46 ± 0.05% and 23.94 ± 4.45% RFP positive cells in the absence or presence of NL4-3 viral infection, an enhancement of 5.37-folds in gene expression due to the presence of Tat and Rev. R2N3-LTR, in contrast, showed only a small proportion 0.25 ± 0.04% of cells to be RFP positive suggesting barely visible gene expression in the absence of Tat and Rev. In the presence of NL4-3 viral infection, there is a significant enhancement of gene expression with 19.33 ± 4.45% cells being RFP-positive with a 76-folds enhancement in transcription. The R2N4-LTR also demonstrated a behavior like that of R2N3-LTR with minimal basal level activity, 0.59 ± 0.07% RFP-positive cells, in the absence of NL4-3 viral infection that enhanced to 18.58 ± 1.09% in the presence of the viral infection with an increase of 31.71-folds in gene expression. Comparable results were obtained when the HIV-1C molecular clone Indie-C1 was used as a source of Tat and Rev (Fig. 2C, right panel). Thus, RBEIII motif duplication reduced the basal level transcriptional activity from both R2N3- and R2N4-LTRs regardless of the copy number difference of the NF-κB sites. In summary, the data are assertive that the RBEIII motif can reduce gene expression noise.

### A homologous RRE element demonstrates a superior reporter response

**Fig. 3:**
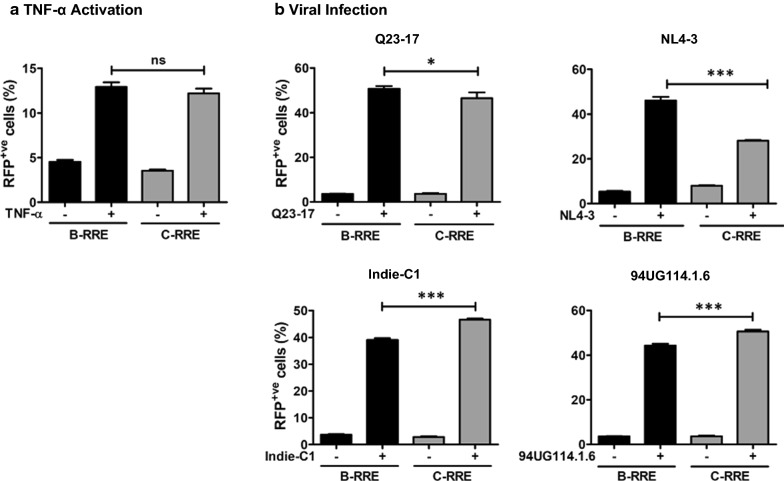
The genetic diversity of RRE may modulate gene expression (**A**) TNF-α activation of cells. CEM-CCR5_LR_ cells harboring RN3 reporter viral vector containing B- or C-RRE element were activated with PMA (5 ng/ml), and the levels of RFP were measured at 24 h by flow cytometry. An unpaired two-tailed *t*-test was performed to compare the data. **B** Viral infection of reporter cells. CEM-CCR5_LR_ cells harboring RN3 reporter viral vector containing B- or C-RRE element were independently infected with one of four viral strains representing different subtypes—Q23-17 (HIV-1A), NL4-3 (HIV-1B), Indie-C1 (HIV-1C), or 94UG114.1.6 (HIV-1D). RFP expression was monitored as described above. An unpaired two-tailed *t*-test was performed to compare the data

The prototype viral vector RN3 used in the above assays contained the RRE element derived from NL4-3, a subtype B molecular clone, but other regulatory elements from the subtype C background, including the viral promoter. To examine the influence of the subtype nature of the RRE, we substituted the RRE of subtype B in the RN3 vector with a homolog RRE derived from Indie-C1 of subtype C (Fig. 1) and compared the two vectors. RN3 vector containing B-RRE or C-RRE demonstrated 4.55 ± 0.22 and 3.54 ± 0.14% cells to be RFP-positive, respectively, in the absence of activation. Both groups of cells showed 2.85- and 3.45-folds enhancement in RFP-positivity, respectively, following TNF-α activation. The difference between the two arms was not significant, suggesting that both the vectors were activated to comparable levels under basal activation conditions (Fig. 3A).

We next asked how the RFP expression was modulated by the presence of B- or C-RRE motifs when the cells were infected with viral strains belonging to different genetic subtypes as the source of Tat and Rev (Fig. 3B). We used four different viral strains, Q23-17, NL4-3, Indie-C1, and 94UG114.1.6 representing subtypes A, B, C, and D, respectively. The data overall appeared to suggest that a homologous regulatory element combination demonstrates significantly superior levels of gene expression. For example, the fraction of RFP positive cells was higher for subtype B viral infection with the vector containing the B-RRE element (46.14 ± 1.63% Vs. 28.12 ± 0.30% for B- and C-RRE, respectively, P < 0.0001). The contrary was true for the HIV-1C viral infection (39.10 ± 0.68% Vs. 46.62 ± 0.42% for B- and C-RRE, respectively, p < 0.0001). Thus, for superior transcriptional activity, a combination of regulatory elements originating from the homologous genetic background may be desirable.

**Fig. 4:**
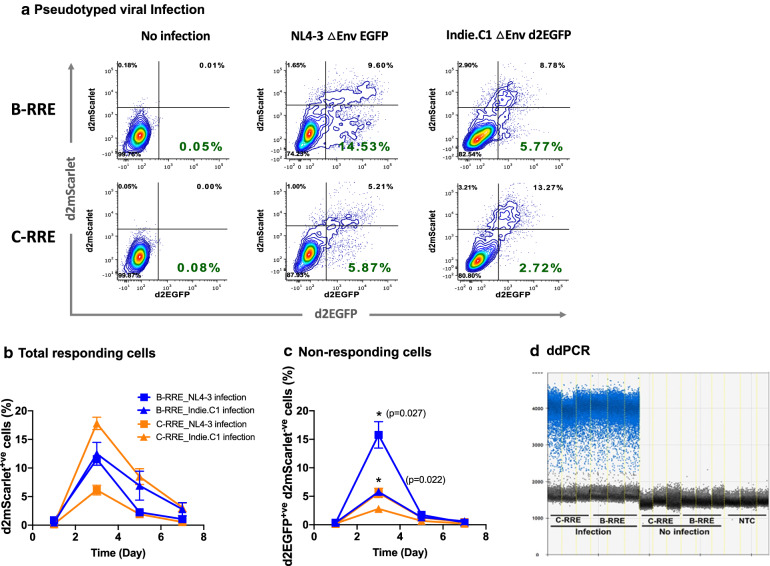
C-RRE exhibits low level gene expression. **A** Longitudinal gene expression analysis. RN3-LTR CEM-CCR5_LR_ cells containing B-RRE or C-RRE were infected with NL4-3_ΔEnv_EGFP or Indie-C1_ΔEnv_d2EGFP molecular clones as a source of Tat and Rev. The expression of the fluorescence proteins was monitored over one week by flow cytometry. The data are representative of three independent experiments. **B** The total percentage of RFP+ve cells and (**C**) Non-responding RFP−ve cells are plotted. Two-way ANOVA with Tukey post-test was used to compare the data. **D** Droplet digital PCR was used to quantify the proviral copy number of Indie-C1_ΔEnv_d2EGFP infection. A two-tailed unpaired t-test (p = 0.14) was used to compare the data

Further, we evaluated the mScarlet expression in a longitudinal analysis to compare the RRE elements of HIV-1B vs. HIV-1C. The CEM-CCR5_RL_ cells were infected with the RN3-B-RRE or RN3-C-RRE viral strains expressing d2mScarlet. Next, the CEM-CCR5_RL_ cells harboring RN3-B-RRE or RN3-C-RRE viral strains were infected with HIV-1B (NL4-3_ΔEnv_EGFP) or HIV-1C (Indie-C1_ΔEnv_d2EGFP) molecular clones pseudotyped with VSV-G as a source of Tat and Rev. The infected cells were monitored for a week for the expression of both the fluorescent proteins by flow cytometry (Fig. 4A). Both the RRE-containing viral strains demonstrated the highest gene expression on day 3 regardless of the subtype nature of Tat and Rev and established latency by day 7 (Fig. 4B). Of note, the proportion of cells infected (GFP^+ve^) but not transcriptionally active (RFP^−ve^) is the lowest when the viruses harbored the C-RRE compared to B-RRE (Fig. 4C). For example, RN3-C-RRE and RN3-B-RRE viral strains co-infected with NL4-3_ΔEnv_EGFP viral clone as a source of Tat and Rev showed 5.87 and 14.53% GFP^+ve^ RFP^−ve^ cells, respectively (Fig. 4A, lower-right quadrants), the difference being significant (p = 0.027). Similarly, the two viral strains co-infected with Indie-C1_ΔEnv_d2EGFP molecular clone demonstrated 2.72 and 5.77% GFP^+ve^ RFP^−ve^ cells, respectively (p = 0.022). The differences were not caused by the differential infectivity rates of the viral strains, as is evident from the analysis of proviral load performed using droplet-digital PCR (Fig. 4D). The B- and C-RRE viral proviral loads were found to be comparable 938.30 ± 15.50 and 1032 ± 85.51, respectively.

### The replication profile of HIV-1 viral strains in R2N3 reporter CMV-CCR5_RL_ cells

**Fig. 5:**
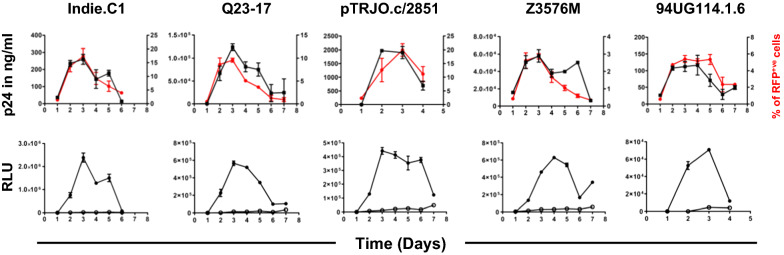
Replication profile of HIV-1 subtypes in R2N3 CEM-CCR5_LR_ reporter cells: R2N3-LTR CEM-CCR5_LR_ cells containing the C-RRE element were independently infected with one of four viral strains representing different HIV-1 subtypes (Q23-17, pTRJO.c/2851, Z3576M, and 94UG114.1.6 representing HIV-1 subtypes A, B, C, and D, respectively). The RFP, Gaussia luciferase, and p24 expression levels were monitored every 24 h for seven days following infection. Each data point represents the mean of three replicate values, and the data were plotted as the mean ± SD. The data are representative of three independent experiments. The closed and open symbols represent viral infection and no infection, respectively

**Fig. 6:**
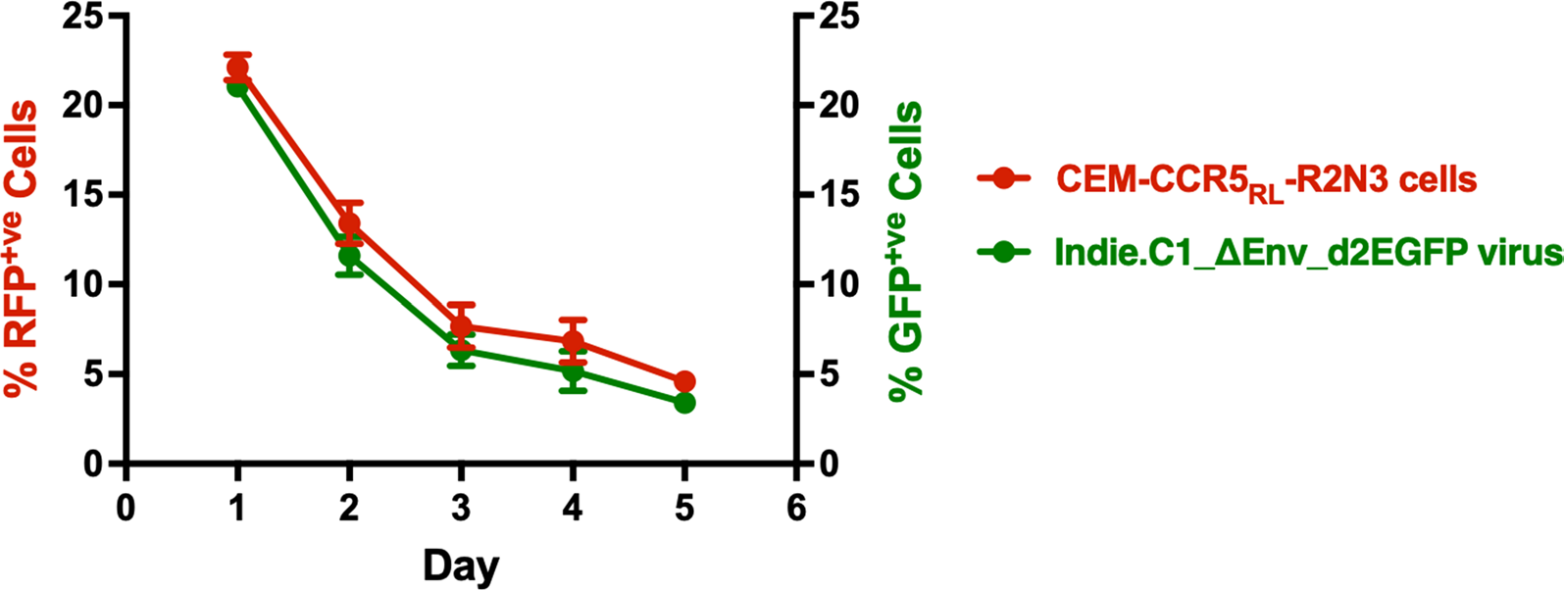
Correlation of reporter activities from CEM-CCR5_RL_ cells infected with the Indie-C1_ΔEnv_d2EGFP virus. The red and green lines represent the activity of the reporter proteins from CEM-CCR5_RL_-R2N3 cells and Indie-C1_ΔEnv_d2EGFP virus, respectively. The data are plotted as the mean percentage value ± SD of RFP and GFP positive cells on the left and right Y-axes, respectively. The kinetics of latency establishment is followed with time. The data are representative of three independent experiments. Two-way ANOVA with Bonferroni post-tests (***p < 0.001 and **p < 0.01) was used for statistical analysis

The data collectively suggested that R2N3-LTR displaying the lowest magnitude of background noise in the absence of activation can serve as a model HIV-1 promoter, especially in combination with homologous C-RRE. To this end, we determined the replication profile of HIV-1 strains representing different viral subtypes. CEM-CCR5_RL_ cells harboring R2N3-LTR were infected with infectious molecular clones Q23-17 (HIV-1A), TRJO.c/2851 (HIV-1B), Z3576M (HIV-1C), and 94UG114.1.6 (HIV-1D) at an MOI of ~ 0.1. The expression of RFP was monitored for a week at 24-h intervals by flow cytometry. Concomitantly, the Gaussia luciferase and p24 antigen levels secreted into the culture supernatant were quantitated (Fig. 5). The replication profiles of the viral strains are consistent in the R2N3 reporter cells regardless of the subtype differences. Importantly, there is a perfect correlation in the expression levels of the two reporter genes in all viral infections. Additionally, the production of the viral p24 antigen also correlated perfectly with that of the reporter genes. All the four viral strains proliferated in CEM-CCR5_RL_ cells, with the reporter genes reaching the peak of expression on day 3 or 4 and falling thereafter. CEM-CCR5_RL_ cells are also conducive to study HIV-1 latency (Fig. 6). When infected with a pseudotyped viral strain Indie-C1_ΔEnv_d2EGFP, CEM-CCR5_RL_ cells were provided for Tat and Rev by trans-complementation. The activity of RFP reporter from CEM-CCR5_RL_ cells correlated with that of the Indie-C1_ΔEnv_d2EGFP virus.

## Discussion

Here, we demonstrated that R2N3-LTR, a variant viral promoter of HIV-1, shows low basal level transcriptional activity in the absence of Tat. HIV-1 LTR is uniquely positioned to centralize the decision-making between the transcriptionally ON and OFF states. The virus integrates into the chromatin, therefore, has access to the transcription machinery of the host. A single viral promoter regulates the expression of all the viral gene products, thus permitting a centralized decision making between the transcription ON and OFF states feasible. Importantly, within the space of a few hundreds of nucleotides, comprising the modulator, enhancer and, basal promoter regions, the viral promoter contains binding sites for several families of transcription factors [21]. The binding sites for NF-κB and Sp-1 are present in multiple copies and tandemly arranged. Given the heavy density of the TFBS arrangement in the viral promoter, these binding sites typically overlap with one another, leading to cooperation and/or competition among transcription factors to bind to the cognate sites leading to transcriptional noise. The transcriptional noise of a typical HIV-1 promoter is significantly higher than that of mammalian promoters and is believed to permit the virus to switch between transcriptionally ON and OFF states [22]. Thus, transcriptional noise of the central transcription circuit of the virus is proposed to be the critical factor in decision making.

In this backdrop, it is noteworthy that at least ten different promoter-variant viral strains have been emerging in India over the past decade. The presence of additional binding sites characterizes the variant promoters for one or more of at least four transcription factor families, including RBF-2, AP-1, LEF-1, and NF-κB [13]. The R2N3-LTR is unique among the emerging variant viral promoters because it contains an additional copy of the RBEIII site, unlike the canonical RN3-LTR and the absence of co-duplication of an NF-κB motif, unlike R2N4-LTR, a different variant viral promoter. Of note, RBEIII motif duplication in HIV-1C contains important differences from that in HIV-1B and typically contains the co-duplication of a complete Ap-1 element [13]. Both RBF-2 and Ap-1 have been implicated in playing a crucial role in establishing and maintaining latency in HIV-1 infection [10, 23]. Based on these studies, we hypothesized and ascertained that the R2N3-LTR is resistant to latency reversal under experimental conditions conducive to activating the canonical RN3-LTR or the variant R2N4-LTR. We propose that the specific configuration of the TFBS in a variant viral strain is likely to impact the latency properties. A detailed investigation is warranted to understand how this process will affect the evolutionary fitness of individual variant viral strains differently.

Further, we propose that a hard-to-activate viral promoter such as the R2N3-LTR can serve as an ideal candidate to develop high throughput screening assays to identify potential latency-reversing agents. Given the considerably low-level gene expression noise compared to the canonical HIV-1 LTRs, the variant R2N3-LTR requires significantly higher-levels of cellular activation for reporter gene expression. One limitation of conventional HTS assays is detecting several false hits due to the overwhelming overlap between the transcription processes of the virus and the host. A stringent HTS assay developed based on a hard-to-activate promoter is likely to minimize this problem and help identify targeted molecules to reverse latency.

## Conclusion

In summary, we partially characterized the activation properties of a variant viral promoter of HIV-1C and found it suitable to examine the mechanisms of viral latency. Additionally, given the low-level gene expression noise, we suggest that viral promoters of this kind can be most suited for developing high throughput screening assays to study HIV latency and formulate cure strategies.

## Materials and methods

### Cell culture

HEK293T and TZM-bl cells were propagated in complete DMEM **(**Gibco™ DMEM, powder, (catalog no. 12800017, Thermo Fisher Scientific, Waltham, USA), supplemented with 250 mM HEPES, (catalog no. TLC021, HiMedia Laboratories, Mumbai, India), sodium bicarbonate (catalog no. S5761, Sigma, St. Louis, USA**)**, 10% fetal bovine serum (catalog no. 10082147, Thermo Fisher Scientific, Waltham, USA), 2 mM l-glutamine (catalog no. TCL012, HiMedia Laboratories, Mumbai, India), 100 units/ml penicillin G sodium salt (catalog no. P3032, Sigma, St. Louis, USA), and 100 μg/ml streptomycin sulfate salt (catalog no. S9137, Sigma, St. Louis, USA). CEM.NKR-CCR5 cells (National Institutes of Health AIDS Research) were grown in complete RPMI medium (RPMI 1640, Catalog no 2340021, Thermo Fisher Scientific, Waltham, USA), supplemented with 10% fetal bovine serum, 2 mM l-glutamine, 100 μg/ml streptomycin, and 100 units/ml penicillin G sodium salt.

### HIV-1 Infectious molecular clones

Various HIV-1 molecular clones representing diverse viral genetic subtypes were procured from the AIDS Reagent Program at the National Institutes of Health, including Q23-17 (HIV-1A, catalog no. 12649), pTRJO.c/2851 (HIV-1B, catalog no. 11747), Z3576M (HIV-1C, catalog no. 13259), and 94UG114.1.6 (HIV-1D, catalog no. 4002).

### Construction of Tat- and Rev-dependent reporter lentiviral vectors

We constructed a panel of four Tat- and Rev- dependent reporter viral vectors co-expressing mScarlet (RFP) and Gaussia Luciferase (G-Luc) under the control of HIV-1C LTR. The RFP contained a degradation domain that reduced the protein half-life to approximately 2 h [17] mScarlet containing a degradation domain (d2mScarlet) is referred to as RFP in this work for simplicity. A single prototype vector RN3 was generated first to construct the two other LTR variant vectors (R2N3 and R2N4) of the panel. In the first step, the segment of pcLGIT [24] containing RRE and EGFP was deleted using the NotI and EcoRI restriction enzyme (RE) sites. A degradation domain of 123 bp amplified from the pCAG-GFPd2 vector (Catalogue no. 14760, Addgene, MA, USA) using a primer pair N4111 and N4112 was substituted into the vector. Concomitantly, we introduced two unique RE sites, AgeI and BlpI, upstream of the degradation domain, using the forward primer N4111 for subsequent manipulation. In the second step, we cloned the mScarlet [25] amplified from the pmScarlet-i_C1 vector (Catalogue no. 85044, Addgene, MA, USA) using a primer pair N4113 and RP N4114 between AgeI and BlpI RE sites. In the third step, we generated the IRES-GLuc cassette using an overlap PCR. In the first PCR, we amplified the IRES element from the pLGIT vector using a primer pair N4412 and N4413. In a second PCR, we amplified the Gaussia Luciferase gene from pCMV-sLuc-IRES-GFP [24] using the primer pair N4414 and N4415. The two PCR products containing an overlap of 48 bp were amplified in a PCR using primers N4412 and N4415. The details of the amplification primers are summarised in Table 1. The final PCR product was cloned between the EcoRI and XhoI RE sites, thus replacing Tat from the parental vector. Lastly, the HIV-1C RRE element from the RN3 vector was replaced by a homolog derived from the pIndie-C1 vector. Using the XhoI and AscI RE sites, the segment comprising of RRE and the splice acceptor 7 (SA7) site was deleted from the RN3 vector, and a homologous region amplified from pIndie-C1 using primers N4230 and N4231 was cloned between the two RE sites directionally. Finally, we replaced the 3' LTR of RN3 vector with variant LTRs of R2N3 and R2N4 amplified from pcLdGIT_R2N3 and pcLdGIT_R2N4 [13] vectors, respectively, to generate the three members of the panel.

**Table 1:** Primers for the construction of lentiviral vectors

Primer name	Primer sequence (5′–3′)
N3028	TTCGACCTTTCTCGAGAGAGATGGTGGTAATAACAACAATGGGTCCG
N3029	CGATTACTTCGGCGCGCCCCCAGAAGTTCCACAATCCTCGTTACAATC
N4111	ACCGCACAGCAAGCGGCCGCCGTCACTAACCGGTGGACATCAAGCTTAGCCATGGCTTCCCGCCGGAGGTGG
N4112	GGGGCGGAATTCGTGACGACTTAATAAACTACACATTGATCCTAGCAGAAGCAC
N4113	CGTCACTAACCGGTATGGTGAGCAAGGGCGAGGC
N4114	AAGCCATGGCTAAGCTTCTTGTACAGCTCGTCCATGCCGCCGGTG
N4230	TTCGACCTTTCTCGAGCATGATGGAGGAATAAAGGAAAATGATACAGAGAATAAGAC
N4231	CGATTACTTCGGCGCGCCAGAAGTTCCACCACTCTCGCTGCC
N4412	AGTTAATTAAGTCGTCACGAATTCCG
N4413	CCTTTGAAAAACACGATGATAATATGGGAGTCAAAGTTCTGTTTGCCC
N4414	GGGCAAACAGAACTTTGACTCCCATATTATCATCGTGTTTTTCAAAGG
N4415	TCCATCATGCTCGAGTTAGTCACC

### Generation and titration of viral stocks

All the viral stocks were generated in HEK293T cells in a six-well tissue culture plate using the standard calcium phosphate method [20]. HEK293T cells (0.5 × 10^6^ cells) were seeded in a 6-well plate one day before the transfection. The cells were transfected with 4 μg of appropriate plasmid DNA, the medium was replenished with complete DMEM 6 h flowing transfection, and the supernatant was collected 48 h post-transfection. To generate pseudotyped lentiviral vector stocks, the cells were co-transfected with a total of 4 μg of plasmid DNA pool consisting of 2 μg of a lentiviral vector, 1 μg of psPax2, 0.70 μg of pHEF-VSVG, and 0.30 μg of pcRev (NIH AIDS reagent program, Catalogue no. 11348, 4693, and11415, respectively). Viral stocks of full-length HIV-1 molecular clones were generated using 4 μg of HIV plasmid DNA along with 0.02 μg of pCMV-GFP plasmid as an internal transfection control. The viral stocks were titered in TZM-bl cells using the Bright-Glo™ Luciferase Assay System (Catalogue. E2610, Promega Corporation, Madison, WI). NL4-3_ΔEnv_EGFP and Indie-C1_ΔEnv_d2EGFP viruses were generated by pseudotyping these viruses with the pHEF-VSVG envelope. The total 4 μg of plasmid DNA pool contained 2.67 μg viral plasmid and a 1.33 μg pHEF-VSVG vector.

### Generation of Tat- and Rev-dependent reporter CEM.NKR-CCR5 cells

CEM.NKR-CCR5 cells were independently infected with the variant viral strains of the panel in a 12-well tissue culture plate. In a volume of 500 μl medium supplemented with 12.5 μg/ml DEAE dextran, 0.3 × 10^6^ CEM.NKR-CCR5 cells were infected at a multiplicity of infection (MOI) of 0.02 to 0.04. Six hours post-transfection, the medium was replaced with 1 ml of complete RPMI medium. Three days following infection, cells positive for red fluorescence were sorted using a flow sorter (Aria III sorter; BD Biosciences, Franklin Lakes, NJ). The infected cells are labeled as CEM-CCR5_RL_ as they co-expressed mScarlet and Gaussia Luciferase in a stable fashion. Sorted cells, 0.5 × 10^6^ CEM-CCR5_RL_ cells/assay, were subjected to different activation conditions—2.5 mM of *N, N′*-Hexamethylene bis(acetamide) (HMBA, Catalogue no. 224235, Sigma, St. Louis, USA), of Trichostatin A (TSA, 100 nM, Catalogue no. T8552, Sigma, St. Louis, USA), TNF-α (10 ng/ml, Catalogue no. 130-094-019, Miltenyi Biotech, Bergisch Gladbach, Germany), PMA (5 ng/ml, Catalogue no. P1585, Sigma, St. Louis, USA), or both TNF-α and PMA. After 24 h of activation, cells were washed with PBS and subjected to live-dead staining using a commercial kit (Catalogue no. L10120, ThermoFisher Scientific, Waltham, USA). An equal number of events (~ 10,000 per assay) were acquired using a flow sorter (BD FACSAria III sorter; BD, Biosciences, NJ, USA). The data were analyzed using the FCS Express6 DeNovo software.

### Infection and replication of viral strains in CEM-CCR5_RL_ cells

For a maximum magnitude of viral infection, we spinoculated CEM-CCR5_RL_ cells in a 48-well tissue culture plate. In CEM-CCR5_RL_ cells, 0.15 × 10^6^ cells per assay in 300 μl of medium supplemented with 12.5 μg/ml DEAE-dextran were deposited in each well and infected with the viral strains independently. The plates were centrifuged at 1000*g* for 2 h at 18 °C; the cells were resuspended in 500 μl of complete RPMI medium and, the cells were incubated for 24 h. The expression of RFP was monitored using a flow cytometer, and the data were analyzed using the FCS Express6 DeNovo software. The levels of p24 and luciferase secreted into the culture medium supernatants were monitored using commercial kits. The p24 levels were measured using a p24 kit (Catalogue no. IR232096, 4th generation p24 ELISA kit; J. Mitra and Co. Pvt. Ltd., New Delhi, India) and those of *Gaussia* luciferase using Pierce™ *Gaussia* luciferase glow assay kit (Catalogue no. 16161, ThermoFisher Scientific).

### Droplet digital PCR (ddPCR)

The two RRE variants of CEM-CCR5_RL_ cells were infected with the Indie-C1_ΔEnv_d2EGFP virus. On Day three post-infection, genomic DNA was isolated from 2 × 10^6^ cells using GenElute Mammalian Genomic DNA Miniprep Kit in 200 µl of elution buffer (Catalogue. G1N350, Sigma, St. Louis, USA). The ddPCR mix of 20 µl consisted of 10 µl 2 × ddPCR™ super mix for probes (Catalogue no. 1863024, Bio-Rad, CA, USA); 900 nM primers (N4262 and N4263, Table 2250 nM probe (N4261, Table 2) and 2 µl of the isolated genomic DNA as a template. Droplets were generated and transferred to a 96-well plate. The PCR conditions were as follows: 95 °C for 5 min, followed by 40 cycles of 94 °C for 15 s, 60 °C for 1 min, and a final 10 min at 98 °C for enzyme deactivation. The droplets were subsequently read automatically by the QX100™ droplet reader (Bio-Rad, CA, USA), and the data were analyzed with the QuantaSoft™ analysis software 1.3.2.0 (Bio-Rad, CA, USA).

**Table 2:** Primers for the digital droplet PCR

Primer name	Primer sequence (5′–3′)
N4261	CATGGCAGGAAGAAGCGGAGACAGCG
N4262	ATCATCCAGGAAGTCAGCCCGAAAC
N4263	TTTGATGATCCTCACTGCTCGGAGG

## Data Availability

The datasets generated for this study are available on a special request to the corresponding author.

## Abbreviations

HIV-1: Human immunodeficiency virus type 1
HIV-1A: HIV-1 subtype A
HIV-1B: HIV-1 subtype B
HIV-1C: HIV-1 subtype C
HIV-1D: HIV-1 subtype D
LTR: Long terminal repeat
TFBS: Transcription factor binding site
RBF-2: Ras response element binding factor 2
RBEIII: RBF-2 binding site
LEF-1: Lymphoid enhancer-binding factor 1
Ap-1: Activator protein 1
NF-κB: Nuclear factor kappa-light-chain-enhancer of activated B cells
NF-AT: Nuclear factor of activated T-cells
Sp1: Specificity protein 1
USF: Upstream transcription factor 1
MFNLP: Most frequent naturally-occurring length polymorphism
TNF-α: Tumor necrosis factor alpha
PMA: Phorbol myristate acetate
TSA: Trichostatin A
HMBA: Hexamethylene bisacetamide
RFP: Red fluorescent protein
G-Luc: Gaussian luciferase
IRES: Internal ribosome entry site
MOI: Multiplicity of infection
RRE: Rev-responsive element
B-RRE: HIV-1B RRE
C-RRE: HIV-1C RRE
CEM-CCR5_RL_: CEM.NKR.CCR5 cells expressing RFP and G-Luc in a Tat and Rev dependent fashion
EGFP: Enhanced green fluorescent protein
d2EGFP: EGFP with degradation domain
d2mScarlet: MScarlet (RFP) with degradation domain
